# GARP Is Regulated by miRNAs and Controls Latent TGF-β1 Production by Human Regulatory T Cells

**DOI:** 10.1371/journal.pone.0076186

**Published:** 2013-09-30

**Authors:** Emilie Gauthy, Julia Cuende, Julie Stockis, Caroline Huygens, Bernard Lethé, Jean-François Collet, Guido Bommer, Pierre G. Coulie, Sophie Lucas

**Affiliations:** 1 WELBIO and de Duve Institute, Université catholique de Louvain, Brussels, Belgium; 2 de Duve Institute, Université catholique de Louvain, Brussels, Belgium; 3 Ludwig Institute for Cancer Research Ltd, Brussels branch, Brussels, Belgium; New York University, United States of America

## Abstract

GARP is a transmembrane protein present on stimulated human regulatory T lymphocytes (Tregs), but not on other T lymphocytes (Th cells). It presents the latent form of TGF-β1 on the Treg surface. We report here that GARP favors the cleavage of the pro-TGF-β1 precursor and increases the amount of secreted latent TGF-β1. Stimulated Tregs, which naturally express GARP, and Th cells transfected with GARP secrete a previously unknown form of latent TGF-β1 that is disulfide-linked to GARP. These GARP/TGF-β1 complexes are possibly shed from the T cell surface. Secretion of GARP/TGF-β1 complexes was not observed with transfected 293 cells and may thus be restricted to the T cell lineage. We conclude that in stimulated human Tregs, GARP not only displays latent TGF-β1 at the cell surface, but also increases its secretion by forming soluble disulfide-linked complexes. Moreover, we identified six microRNAs (miRNAs) that are expressed at lower levels in Treg than in Th clones and that target a short region of the *GARP* 3’ UTR. In transfected Th cells, the presence of this region decreased GARP levels, cleavage of pro-TGF-β1, and secretion of latent TGF-β1.

## Introduction

Regulatory T cells (Tregs) are a subset of CD4^+^ T lymphocytes. Tregs negatively regulate immune responses [1]. They prevent auto-immune pathology by suppressing the activity of self-reactive T cells. Their development and function require transcription factor FOXP3, which is encoded on chromosome X. Males carrying a mutated *FOXP3* allele show a profound Treg deficiency and a severe autoimmune syndrome.

On the other hand, excessive Treg function favors cancer progression in mice, as prophylactic or therapeutic depletion of Tregs induced regression of transplanted tumors by improving anti-tumor T cell responses [2–6]. There is accumulating evidence that Tregs contribute to cancer progression also in humans [7,8].

Therapeutic targeting of Tregs could therefore prove beneficial in human pathologies. However, the immunosuppressive mechanisms of human Tregs have not been well characterized, in part because of the difficulty to identify these cells without ambiguity. To circumvent this problem, we derived stable clones of human Tregs, defined by the presence of demethylated CpG dinucleotides in the first intron of the *FOXP3* gene [9]. This epigenetic modification is the most specific marker of Tregs in human hematopoietic cells [10–12]. We used these clones to show that Tregs, but not other T lymphocytes, produce the active form of TGF-β1 after T cell receptor (TCR) stimulation [9].

**Figure 1:**
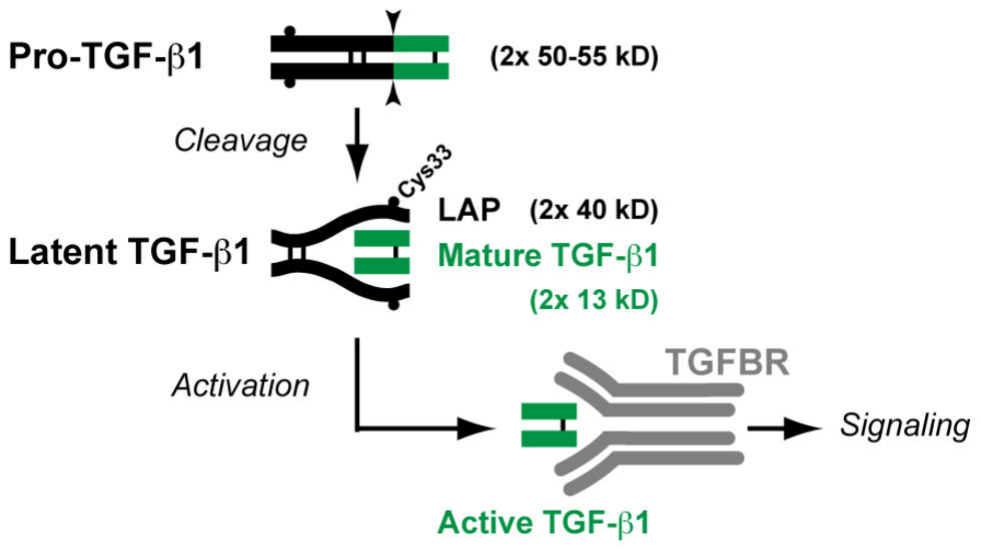
Schematic representation of TGF-β1 processing. Sites of proteolytic cleavage are indicated by arrow heads. Thin black bars indicate disulfide bonds. Small black circles indicate cysteine 33 (Cys33). This position is relative to the starting methionine of the *TGFB1* translation product. It must be noted that some authors number this cysteine as Cys4, referring to the position in pro-TGF-β1 after cleavage of the signal peptide. LAP: Latency Associated Peptide. TGFBR: TGF-β receptors.

TGF-β1 is a potent immunosuppressive cytokine in mice, as best illustrated by the severe autoimmune phenotype of the *Tgfb1* knock-outs [13]. *In vitro*, it affects the proliferation, differentiation and function of many human immune cell types [14]. Its production is a tightly regulated multi-step process that is very similar in humans and mice (Figure 1 and ref [15].). The precursor named pro-TGF-β1 homodimerizes prior to cleavage by pro-protein convertase FURIN. The resulting product is called latent TGF-β1, in which the C-terminal fragment, or mature TGF-β1, remains non-covalently bound to the N-terminal fragment known as the Latency Associated Peptide, or LAP (Figure 1). This latent complex is inactive because LAP prevents mature TGF-β1 from binding to its receptor. Further processing, commonly referred to as “latent TGF-β1 activation”, is required to release mature TGF-β1 from LAP.

Most immune cells, including CD4^+^ and CD8^+^ T lymphocytes with or without stimulation, secrete soluble latent TGF-β1 [9,16,17]. After TCR stimulation, Tregs bear latent TGF-β1 on their surface [18,19]. This occurs through binding to GARP [17,20], a transmembrane protein with a large extracellular domain containing 20 leucine-rich repeats. GARP protein was found after TCR stimulation in human Tregs, but not in other T lymphocytes [17,20–22], explaining why only Tregs display latent TGF-β1 on their surface. What is still not known is how stimulated Tregs activate latent TGF-β1. Activation occurs close to the Treg surface because active TGF-β1 is not detected in the supernatants but exerts its paracrine actions when Tregs contact target cells [9]. Forced expression of GARP is sufficient to induce latent TGF-β1 binding to the cell surface, but is not sufficient to induce active TGF-β1 production [17]. Whether GARP solely functions as a receptor for latent TGF-β1, or whether it plays additional roles in the production of the cytokine in T cells has not been shown.

Recently, a disulfide bridge implicating Cys33 in the LAP dimer was shown to link latent TGF-β1 to GARP in transfected 293 cells [23]. Inside human platelets and fibroblasts, Cys33 bridges latent TGF-β1 to other proteins, called LTBPs (Latent TGF-β Binding Proteins). LTBPs are large proteins that are secreted in the extracellular matrix. Disulfide bonding to LTBPs facilitates folding and secretion of latent TGF-β1, and could also be required for TGF-β1 activation by some cell types [24,25].

Here, we analyzed whether GARP regulates the production of TGF-β1 in human T cells, in a manner similar to LTBPs in other cell types. We found that GARP increases the cleavage of the pro-TGF-β1 precursor and induces production of soluble GARP/latent TGF-β1 disulfide-linked complexes by human Tregs. In our effort to understand GARP regulation, we also sought to determine whether GARP levels are regulated post-transcriptionally by miRNAs. We identified 6 miRNAs expressed in human T cells that decrease GARP levels and hence, production of membrane-bound and secreted latent TGF-β1.

## Results

### GARP facilitates cleavage of pro-TGF-β1 and secretion of latent TGF-β1 in human T cells

**Figure 2:**
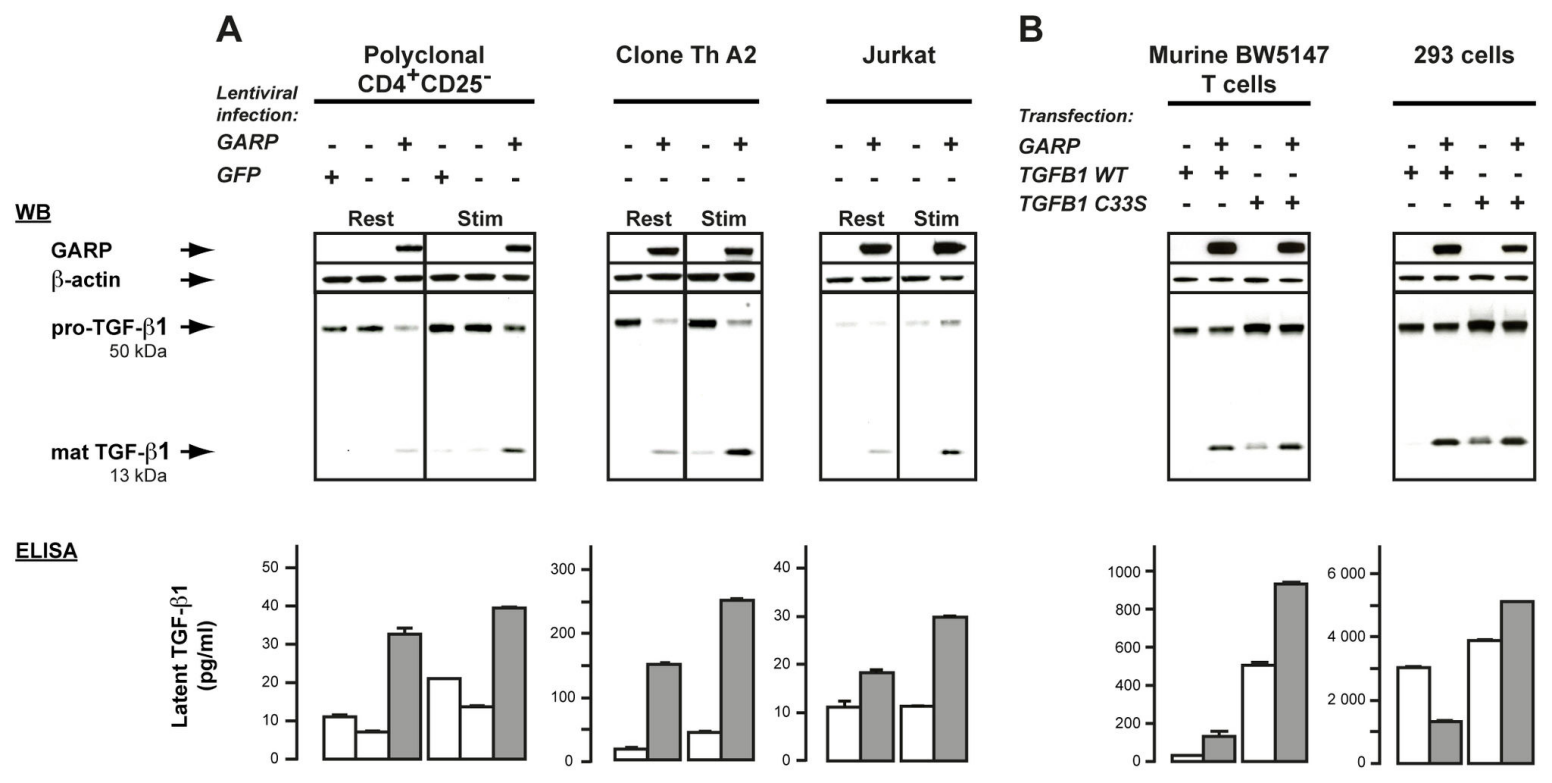
GARP increases cleavage of the pro-TGF-β1 precursor and secretion of latent TGF-β1 in T lymphocytes. Cell lysates were analyzed by WB after SDS-PAGE under reducing conditions with antibodies against GARP, β-actin and a C-terminal epitope of the TGF-β1 peptide (top panels). Supernatants were treated or not with acid and analyzed by ELISA to measure concentrations of total (latent + active) and active TGF-β1, respectively (bottom panels). Total TGF-β1 detected in the acid-treated samples corresponds to latent TGF-β1 because no active TGF-β1 was detected in the non-treated samples. Values represent means of duplicates + SD. **A**. Analysis of human T cell lines transduced or not with lentiviruses coding GARP or GFP. T cells were left resting (Rest) or stimulated for 24 hours with anti-CD3/CD28 antibodies (Stim) in serum-free medium. **B**. Analysis of stable clones of murine BW5147 T cells and 293 cells transiently transfected with *GARP* and WT or C33S mutant *TGFB1*. Untransfected BW5147 and 293 cells express low levels of endogenous TGF-β1 that are not detectable by WB in these conditions (not shown). By comparison to WT, transfection of mutant C33S results in increased production of total TGF-β1 (pro- + mature), as previously described [49].

**Figure 3:**
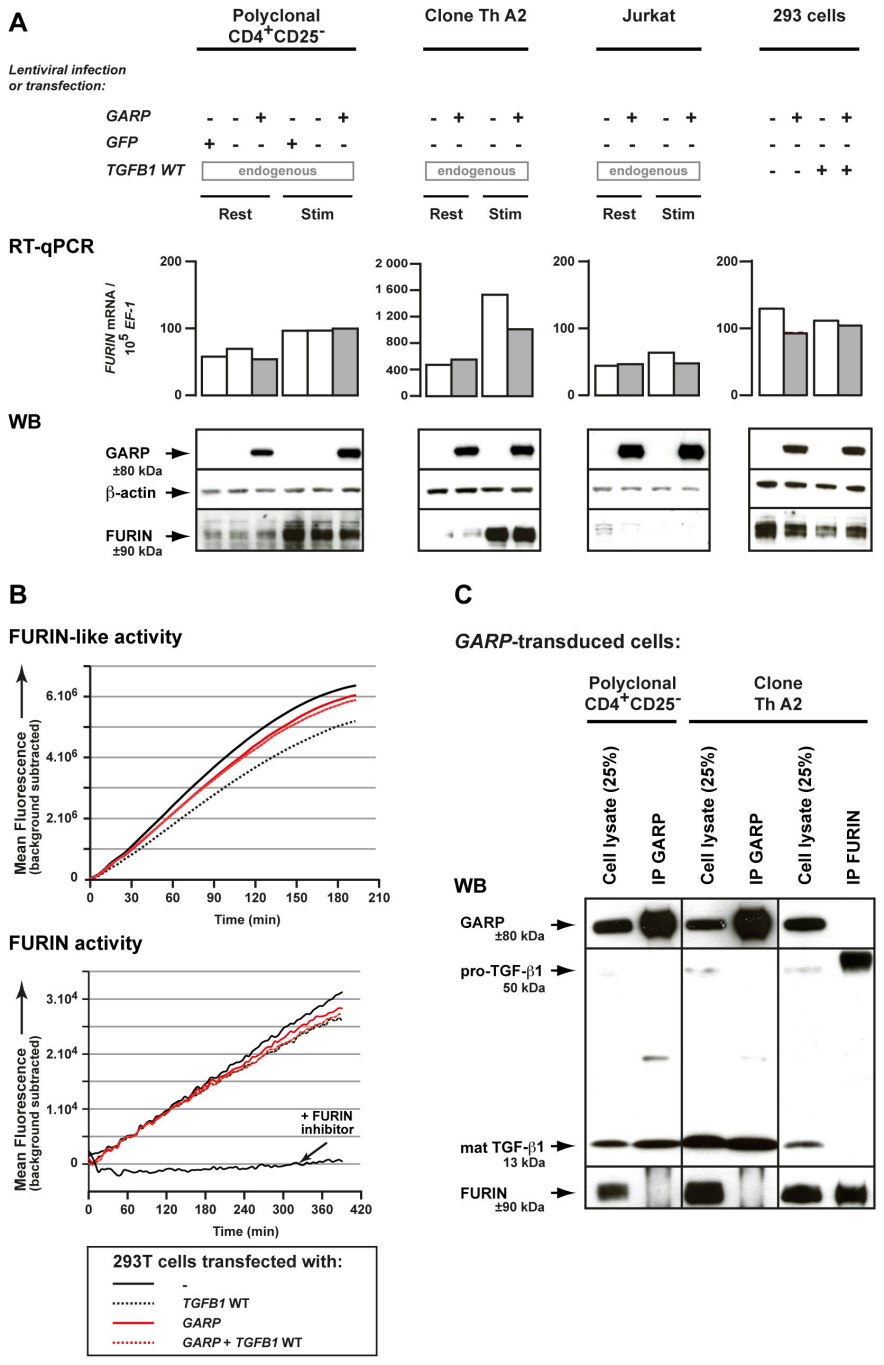
GARP does not increase FURIN expression or activity, and does not co-immunoprecipitate with FURIN. **A**. Expression of FURIN mRNA and protein were analyzed by RT-qPCR and WB in the human cells described in Figure 2. **B**. FURIN activity was measured 24 hours after transfection of 293 cells. Lysates of transfected cells were incubated with a FURIN fluorogenic substrate directly (top panel), or after capture on plastic-coated anti-FURIN antibody (bottom panel), to measure FURIN-like or FURIN specific activity, respectively. Graphs show mean fluorescence intensity at the indicated time (min) after addition of the substrate. The FURIN inhibitor Dec-RVKR-CMK was added to one condition to verify the specificity of the assay. **C**. Lysates of cells described in Figure 2 were immunoprecipitated with anti-GARP (IP GARP) or anti-FURIN (IP FURIN) antibodies. Immunoprecipitation products or total cell lysates (25% of input used for IPs) were analyzed by WB with anti-GARP, anti-TGF-β or anti-FURIN antibodies, as indicated.

We used lentiviruses to transduce *GARP* into GARP^-^ human CD4^+^ T cells, more precisely polyclonal CD4^+^CD25^-^ cells, a CD4^+^ Th clone and Jurkat cells [17]. We first examined cleavage of pro-TGF-β1 by western blot (WB) after SDS-PAGE under reducing conditions. We used an antibody directed against a TGF-β1 C-terminal epitope that detects uncleaved pro-TGF-β1 monomers as ±50 kDa bands and monomers of the mature cleaved cytokine as ±13 kDa bands. With this reagent, increased precursor cleavage should decrease the intensity of the 50 kDa band and increase that of the 13 kDa band. As shown in Figure 2A (top panels), lentiviral-mediated GARP expression increased precursor cleavage in all T cell lines tested at rest or after TCR stimulation. This increased cleavage might involve FURIN, the pro-protein convertase that cleaves pro-TGF-β1 in many cell types [15]. However, we observed no increase in FURIN mRNA or protein levels, nor in FURIN activity, in *GARP* transfected cells (Figure 3A and 3B). We also failed to detect a GARP-FURIN interaction in co-immunoprecipitation experiments (Figure 3C).

Next, we measured the TGF-β1 secreted by the *GARP*-transduced T cells. As expected, active TGF-β1, i.e. LAP-free mature TGF-β1, was undetectable in the supernatants. Latent TGF-β1 was secreted by the three non-transduced T cell lines at various levels which in most cases increased upon TCR stimulation (Figure 2A, bottom panels). Lentiviral-mediated GARP expression increased latent TGF-β1 secreted by all T cell lines, both at rest (1.6 to 9.2 fold) and after TCR stimulation (2.6 to 5.7 fold).

Altogether, our data show that in human T cells, GARP facilitates the cleavage of pro- TGF-β1 and the subsequent secretion of latent TGF-β1. As lentiviral-mediated GARP expression was not sufficient to induce active TGF-β1 production [17], we could not examine the TGF-β1 activation step in this model.

### GARP is disulfide bonded to Cys33 of latent TGF-β1 in T cells

The above results in human T cells contrast with those obtained in 293 cells, in which induction of GARP expression was recently shown to reduce TGF-β1 secretion [23]. This was proposed to result from tethering of the cytokine at the cell surface through disulfide bonding between GARP and Cys33 in TGF-β1. We thus examined if the two proteins are disulfide bonded also in T cells.

**Figure 4:**
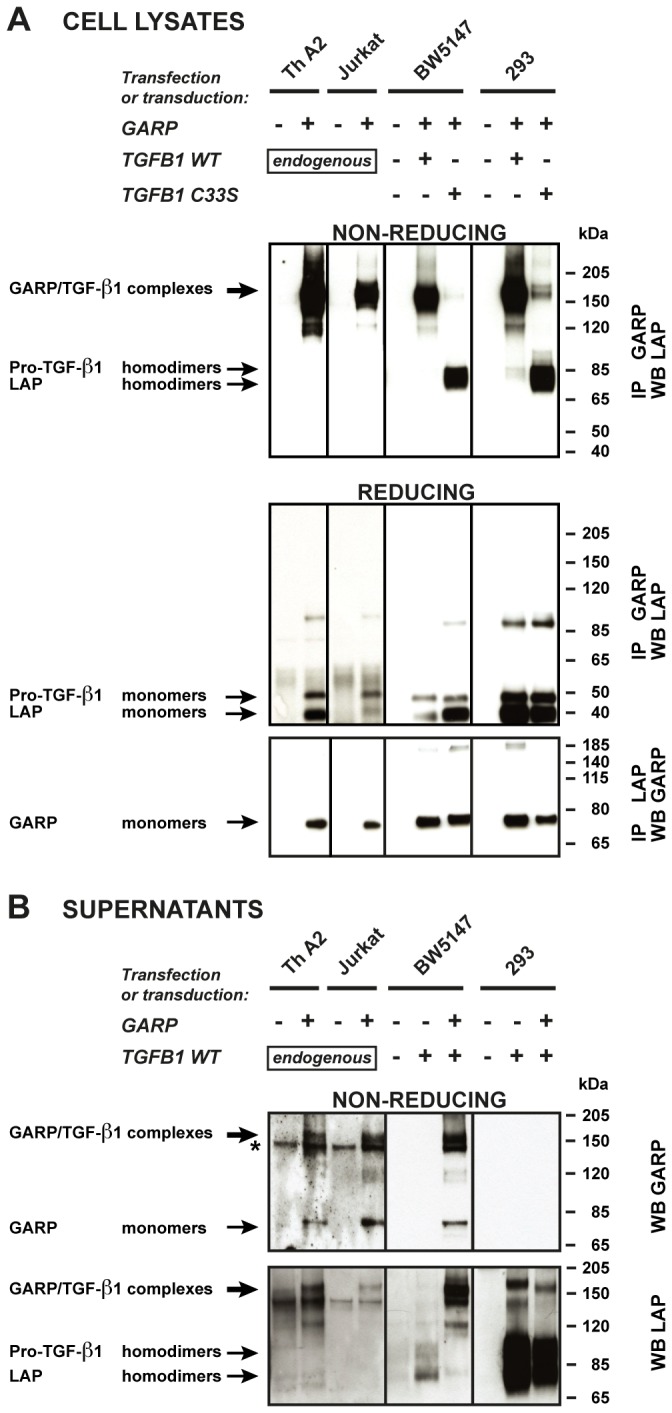
Disulfide-linked GARP/TGF-β1 complexes are released in the supernatant of T cells, but not 293 cells. **A**. Cells described in Figure 2 were lysed and immunoprecipitated (IP) with anti-GARP or anti-LAP antibodies. IP products were submitted to SDS-PAGE under non-reducing or reducing conditions, followed by WB with anti-LAP antibodies (top and middle panels), or anti-GARP antibodies (bottom panels). Pro-TGF-β1 and LAP homodimers in the top panels are not clearly resolved, but can be distinguished better with longer migrations or higher concentrations of polyacrylamide. The +/- 85-90 kDa bands that appear in the middle panel correspond to non-specific bands, or to incompletely reduced pro-TGF-β1. **B**. Cells (2x10^6^/ml for murine and human T cells, 2.5x10^5^/ml for transfected 293 cells) were incubated in serum free medium during 24 hours. Different cell concentrations were used to adjust for the different amounts of secreted TGF-β1 (see Figure 2). Human Th A2 and Jurkat cells were stimulated with anti-CD3/CD28 antibodies to increase secretion. Supernatants (0.5-10 µl) were analyzed by WB under non-reducing conditions with anti-GARP and anti-LAP antibodies. * Band that also appears when the secondary anti-IgG2b-HRP antibody is used alone (without anti-GARP antibody), due to cross reactivity against the anti-CD3/CD28 antibodies used for T cell stimulation.

GARP/TGF-β1 complexes were immunoprecipitated (IP) with anti-GARP antibodies, submitted to SDS-PAGE under non-reducing conditions, and analyzed by WB with anti-LAP antibodies (Figure 4A, top panel). GARP/TGF-β1 complexes predominantly appeared as high molecular weight bands (±150 kDa) in two human T cell lines (clone Th A2 and Jurkat) transduced with *GARP*, suggesting covalent linkage between GARP and TGF-β1 also in T cells. To confirm that this occurs through disulfide bonding to Cys33 in TGF-β1, we transfected human *GARP* and wild type (WT) or C33S mutant *TGFB1* in murine BW5147 lymphoma T cells and in 293 cells as controls. We used BW5147 T cells because they can be transfected with much higher efficiency than the human T cell lines used above. High molecular weight GARP/TGF-β1 complexes (±150 kDa) were immunoprecipitated from BW5147 T cells and 293 cells transfected with *GARP* and WT *TGFB1*. In contrast, they were not immunoprecipitated from BW5147 T cells and 293 cells transfected with *GARP* and the C33S *TGFB1* mutant. For these cells, only homodimers of pro-TGF-β1 and homodimers of LAP were obtained, indicating that GARP still interacts with pro- and latent TGF-β1 but not covalently when Cys33 is mutated to Ser. After SDS-PAGE under reducing conditions, GARP/TGF-β1 complexes immunoprecipitated with anti-GARP or anti-LAP antibodies were disrupted into bands corresponding to monomers of pro-TGF-β1 and LAP (Figure 4A, middle panel), and monomers of GARP (Figure 4A, bottom panel). All these results demonstrate that GARP is disulfide-linked to Cys33 of TGF-β1 in human and murine T cells, like it is in 293 cells.

As additional controls (Figure 2B and Figure S1), we verified that *GARP* transfection in BW5147 T cells increased cleavage, surface binding and secretion of latent TGF-β1, like in human T cells, whereas in 293 cells, it increased cleavage and surface binding but decreased secretion of latent TGF-β1, as reported [23]. Interestingly, GARP increased cleavage and surface binding of both WT and C33S mutated TGF-β1 in the two cell types, indicating that Cys33 is not required for these GARP-mediated functions.

Altogether, we conclude that disulfide linkage to GARP prevents latent TGF-β1 secretion in 293 cells, but not in human or murine T cells. On the contrary, in T cells TGF-β1 secretion is increased in the presence of GARP, in spite of disulfide-linkage.

### Latent TGF-β1 secretion in the presence of GARP appears to occur through shedding of GARP/TGF-β1 complexes from T cell membranes

A plausible explanation to our observations is that GARP/TGF-β1 complexes are shed from the surface of T cells but not of 293 cells. In support of this hypothesis, we detected high molecular weight complexes (±150 kDa) containing both GARP and LAP in the culture medium of all GARP-transfected T cells tested, namely human Th A2 and Jurkat cells and murine BW5147 T cells (Figure 4B, top and bottom panels). The products secreted by GARP-expressing 293 cells were different: no high molecular weight complexes detected with the anti-GARP antibody (Figure 4B, top panel) and abundant pro-TGF-β1 and LAP homodimers detected with the anti-LAP antibody (Figure 4B, bottom panel).

These results indicate that the increased secretion of latent TGF-β1 in *GARP*-transfected human and murine T cells results from shedding of GARP/TGF-β1 complexes from the cell surface. This process may be restricted to T lymphocytes, as it is not observed in transfected 293 cells.

### GARP/TGF-β1 complexes are produced by human Tregs, which naturally express GARP

We next verified if disulfide-linked GARP/TGF-β1 complexes were also produced by stimulated human Tregs, which naturally express GARP [17,20]. For this, we used six types of human CD4^+^ T cells, in which we evaluated the proportion of Treg cells by quantification of demethylated *FOXP3i1* sequences: a/ CD4^+^CD25^+^CD127^lo^ “Treg” cells (26-96% Tregs) and CD4^+^CD25^-^CD127^hi^ Th cells (<1% Tregs) analyzed immediately after sorting from PBMCs; b/ sorted CD4^+^CD25^+^CD127^lo^ and CD4^+^CD25^-^CD127^hi^ cells amplified *in vitro* during 12-14 days (17-52% and <1% Tregs, respectively); and c/ clonal populations of Treg cells (pure Tregs) and Th cells (pure Th) that we described previously [9]. As expected, the three types of Treg but not Th cells expressed GARP after TCR stimulation (Figure S2).

**Figure 5:**
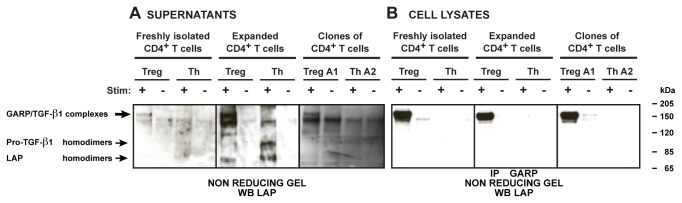
Disulfide-linked GARP/TGF-β1 complexes are released by stimulated human Tregs, which naturally express GARP. The indicated Treg and Th cell populations were left resting or stimulated with anti-CD3/CD28 antibodies in serum-free medium. **A**. Supernatants were collected after 48 hours. **B**. Cell lysates were collected after 24 hours and IP with an anti-GARP antibody. Supernatants (A) and immunoprecipitated lysates (B) were submitted to SDS-PAGE under non-reducing conditions, then analyzed by WB with anti-LAP antibodies. Similar results were obtained with freshly isolated CD4^+^ T cells from 2 other donors and with expanded CD4^+^ T cells from 5 others donors.

With the anti-LAP antibody, we detected high molecular weight complexes (±150 kDa) in the culture medium of the three types of stimulated Tregs (Figure 5A). No such complexes were detected in the supernatants of Th cells, some of which contained high levels of pro- TGF-β1 and LAP homodimers (Figure 5A). Similar high molecular weight complexes were also detected in the lysates of the stimulated Tregs after immunoprecipitation with anti-GARP antibodies, demonstrating that they contained both GARP and TGF-β1 (Figure 5B).

We concluded that in human Tregs, GARP expression upon TCR stimulation induces the release of a previously unknown form of soluble latent TGF-β1 that is disulfide-linked to GARP.

### *miR-142-3p*, *miR-181a*, *b*, *c* and *d* and *miR-185* target the 3’ UTR of the GARP mRNA

**Figure 6:**
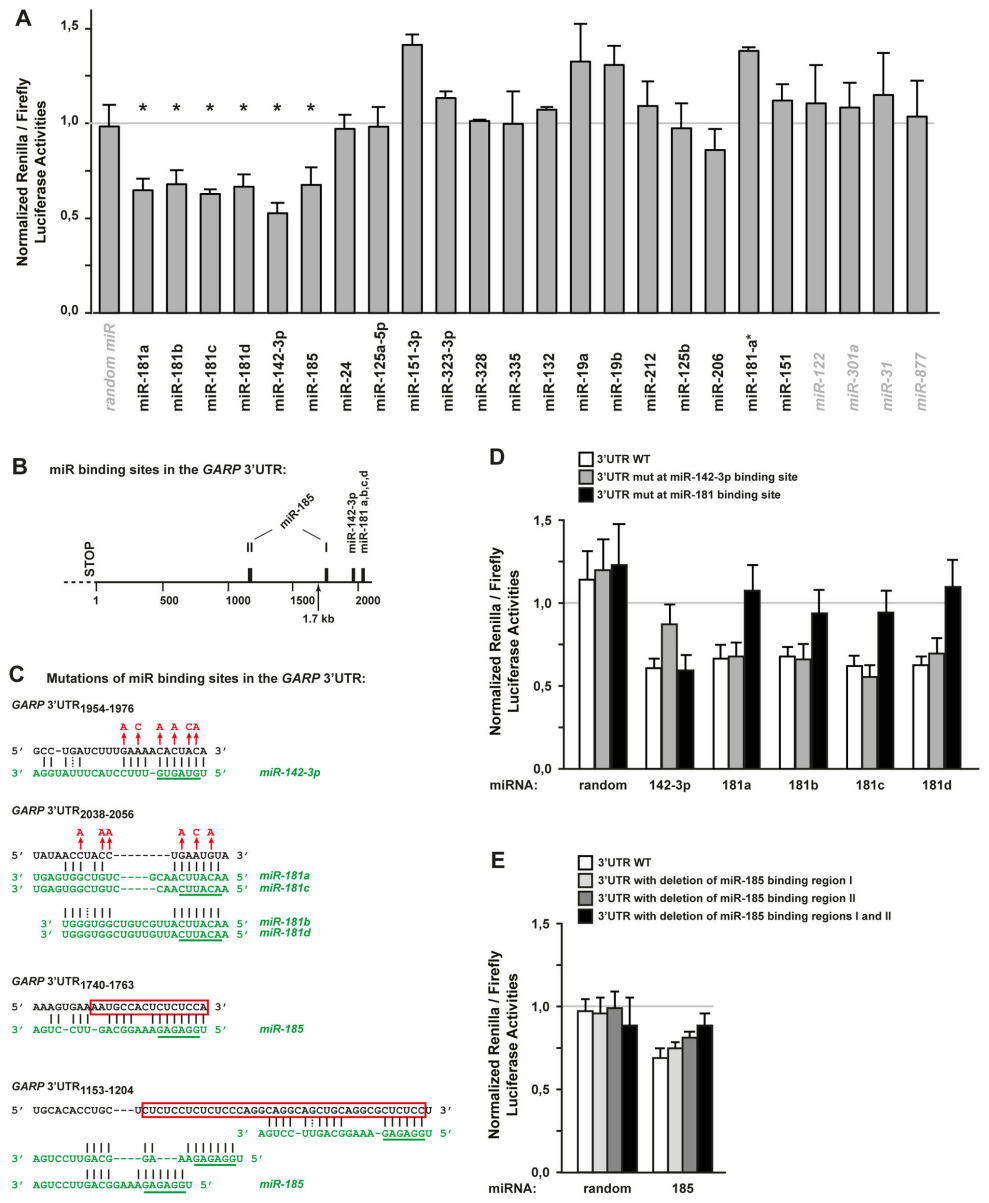
Identification of 6 miRNAs targeting the *GARP* 3’ UTR in 293 cells. **A**. 293 cells were cotransfected with a reporter plasmid and the indicated miRNA mimics (black regular: 20 miRNAs predicted to bind the *GARP* 3’ UTR and expressed in T cells; grey italic: negative controls). The reporter plasmid contains the *GARP* 3’ UTR cloned downstream of the Renilla luciferase gene, and a Firefly luciferase gene to control for transfection efficiency. Graphs indicate the ratio of Renilla to Firefly activities in cotransfected cells, normalized to the ratio in cells transfected with the plasmid alone (no miRNA). Data presented are the mean normalized ratios + SD measured in 3 to 12 independent experiments. * p < 0,0001 by comparison to control random miRNA for normalized ratios <1 (unpaired two-tailed Student’s t test). **B**. Schematic representation of the *GARP* 3’ UTR region, with predicted miRNA binding sites indicated by black boxes. The end of the truncated 1.7 kb 3’ UTR region cloned in a lentivirus used in Figure 9 is indicated by an arrow. **C**. Nucleotide sequences in black correspond to regions of the *GARP* 3’ UTR where the indicated miRNAs are predicted to bind (subscript numbers indicate positions relative to the first nucleotide after the STOP codon). miRNA sequences are shown in green, with seed regions underlined. Optimal alignments between the miRNA and the partial *GARP* 3’ UTR sequences were calculated with the mfold software [50]. Red letters and red boxes indicate nucleotides in the *GARP* 3’ UTR that were substituted or deleted by targeted mutagenesis, respectively. **D** and **E**. 293 cells were cotransfected as in A, except that the reporter plasmid contained wild type (WT) or mutated (mut) forms of the *GARP* 3’ UTR, as indicated. Data presented are the normalized ratios of Renilla to Firefly activities (means of triplicates + SD) and are representative from 2 to 4 independent experiments.

Because GARP appears to regulate TGF-β1 secretion by T cells and TGF-β1 is important for the suppressive function of Treg cells, we sought to identify mechanisms that control GARP expression. We showed previously that some human Th clones expressing high levels of the *GARP* mRNA did not contain detectable GARP protein [17]. We examined whether miRNAs contribute to the post-transcriptional regulation of GARP levels. Using 4 publicly available bioinformatics programs, we identified 41 human miRNAs predicted to target the 3’ UTR of the *GARP* mRNA (Table S1). Expression of 20 of the 41 miRNAs was detected in human non-regulatory T cells by microarray analysis ( [26] and Table S1). We tested these 20 miRNAs by cotransfecting into 293 cells miRNA mimics and a reporter plasmid containing the *GARP* 3’ UTR downstream of a Renilla luciferase sequence. Six miRNAs, namely *miR-181a*, *b*, *c* and *d*, *miR-142-3p* and *miR-185*, significantly decreased the reporter’s expression (Figure 6A). These 6 miRNAs also decreased GARP protein levels in 293 cells co-transfected with the *GARP* full-length cDNA (Figure S3).

To determine whether targeting by the 6 miRNAs is direct, we mutated the miRNA predicted binding sites in the *GARP* 3’ UTR (Figure 6B and 6C). *miR-181a* to *d* have very similar sequences and are predicted to bind to a single site in the *GARP* 3’ UTR, close to the predicted *miR-142-3p* binding site (Figure 6B). Mutation of the *miR-142-3p* binding site, but not that of the *miR-181* site, suppressed the ability of the *miR-142-3p* mimic to decrease reporter activity in 293 cells (Figure 6D). Conversely, mutation of the *miR-181* site, but not that of the *miR-142-3p* site, suppressed the ability of the four m*iR-181* family members to decrease reporter activity (Figure 6D). For *miR-185*, microRNA.org predicts binding at a site annotated as region “I” in Figure 6B, and we identified manually other putative binding sites in indicated region “II”. Deletion of either region alone had mild effects, but deletion of both regions restored reporter activity to that observed in cells transfected with a scramble control miRNA (Figure 6E). Taken together, our results indicate that the *GARP* 3’ UTR is a direct target of 6 miRNAs expressed in human T cells.

### *miR-142-3p*, *miR-181a* and *miR-185* decrease GARP protein levels when overexpressed in polyclonal populations enriched in Tregs

**Figure 7:**
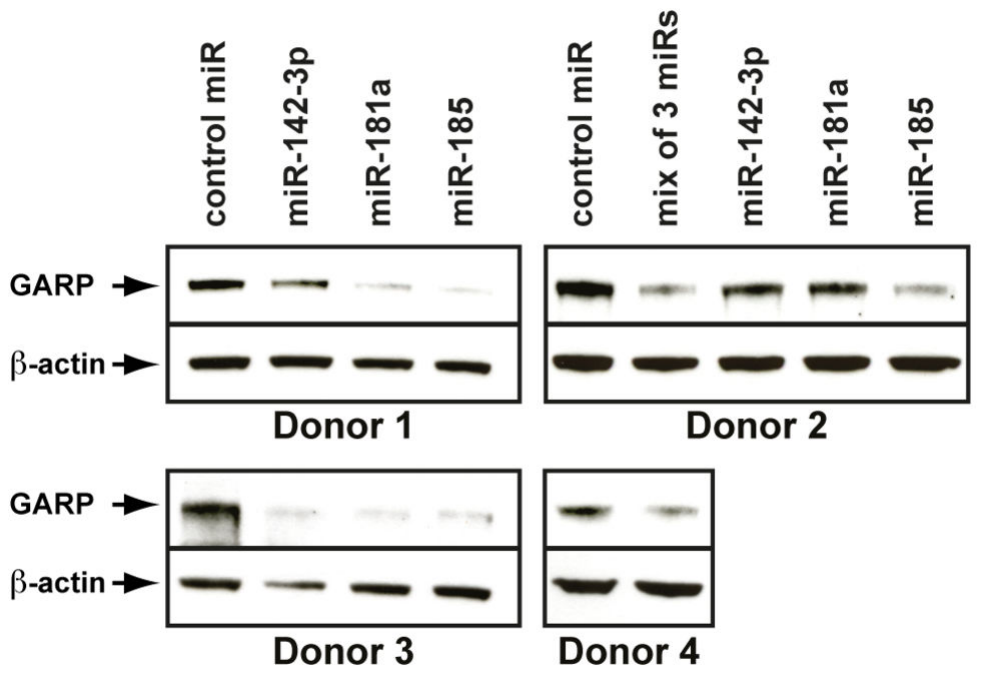
Endogenous GARP levels in Tregs are reduced after transfection of *miR-181a*, *miR-142-3p* and *miR-185* mimics. Polyclonal CD4^+^CD25^+^CD127^lo^ populations were purified from human PBMCs and amplified *in* *vitro*. Amplified cells were electroporated with the indicated miRNA mimics and stimulated 6 hours later with anti-CD3/CD28 antibodies. Cell lysates were collected 24 hours later and analyzed by WB with anti-GARP and anti-β-ACTIN antibodies.

To test whether these miRNAs can control endogenous GARP levels in human T cells, we transfected miRNA mimics in T cell populations with endogenous GARP expression. Because freshly isolated Tregs (CD4^+^CD25^+^CD127^lo^ cells) or Treg clones cannot be transfected efficiently in our hands, we used CD4^+^CD25^+^CD127^lo^ lymphocytes that had been amplified *in vitro*, as described above. These expanded polyclonal populations enriched in Tregs were electroporated with miRNA mimics and stimulated with anti-CD3/CD28 antibodies to induce GARP expression. GARP protein was decreased after transfection with *miR-142-3p*, *miR-185*, *miR-181a*, or a mixture of the three, by comparison to a control miRNA (Figure 7).

### *miR-142-3p*, *miR-181a*, *b*, *c* and *d* and *miR-185* are expressed at higher levels in human CD4^+^ Th clones than in Treg clones

**Figure 8:**
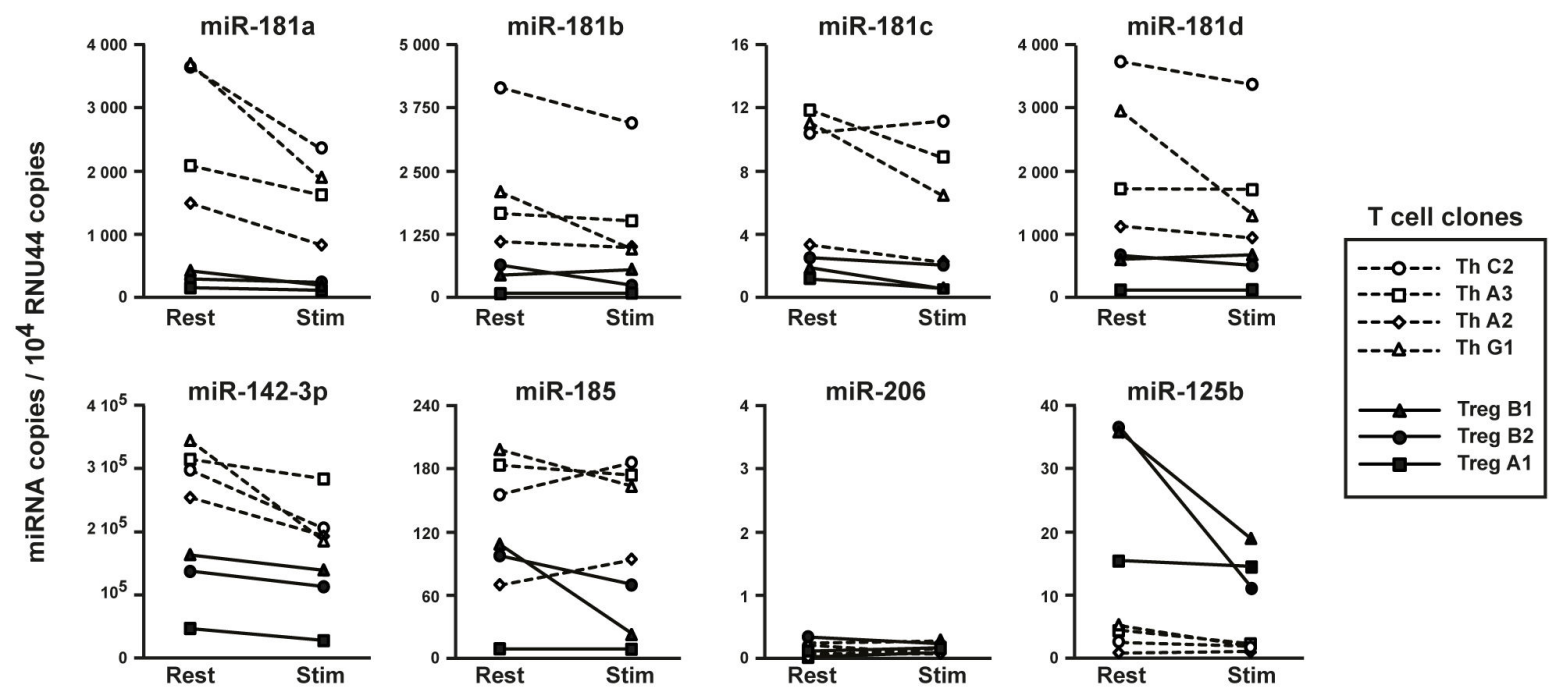
Expression of miRNAs in human CD4^+^ Th and Treg clones. Expression levels of the indicated miRNAs and of control small nuclear RNA RNU44 were measured by RT-qPCR in 4th clones (dashed lines) and 3 Treg clones (plain lines), at rest or 24 hours after stimulation with anti-CD3 and anti-CD28 antibodies.

If the 6 miRNAs identified above were to play a role in regulating GARP protein levels in human T cells, we expected their expression to be higher in Th than in Treg clones. RT-qPCR analysis indicated that *miR-142-3p* was expressed at the highest overall levels, and in average 2.5 times more in Th than in Tregs (Figure 8). *miR-181a*, *b* and *d* were expressed at intermediate levels, and 4.8 to 9.6 times more in Th than in Treg clones. Finally, *miR-185* and *miR-181c* were expressed at lower levels, and 2.9 to 5.7 times more in Th than in Treg clones. *miR-206*, which slightly decreased luciferase activity but not GARP protein in 293 cells (Figure 6A and Figure S3), was barely detectable in most clones. Negative control *miR-125b* was expressed at higher levels in Treg than in Th clones. Thus, the expression profiles of the 6 miRNAs are compatible with a role in post-transcriptional control of GARP levels in human T cells.

### *GARP* 3’ UTR controls GARP protein levels and amounts of latent TGF-β1 secreted by a human CD4^+^ Th clone

We examined whether endogenous miRNAs played a role in silencing GARP expression in human Th cells. We failed to reproducibly induce GARP protein in Th cells by transfection with specific miRNA inhibitors (

**Figure 9:**
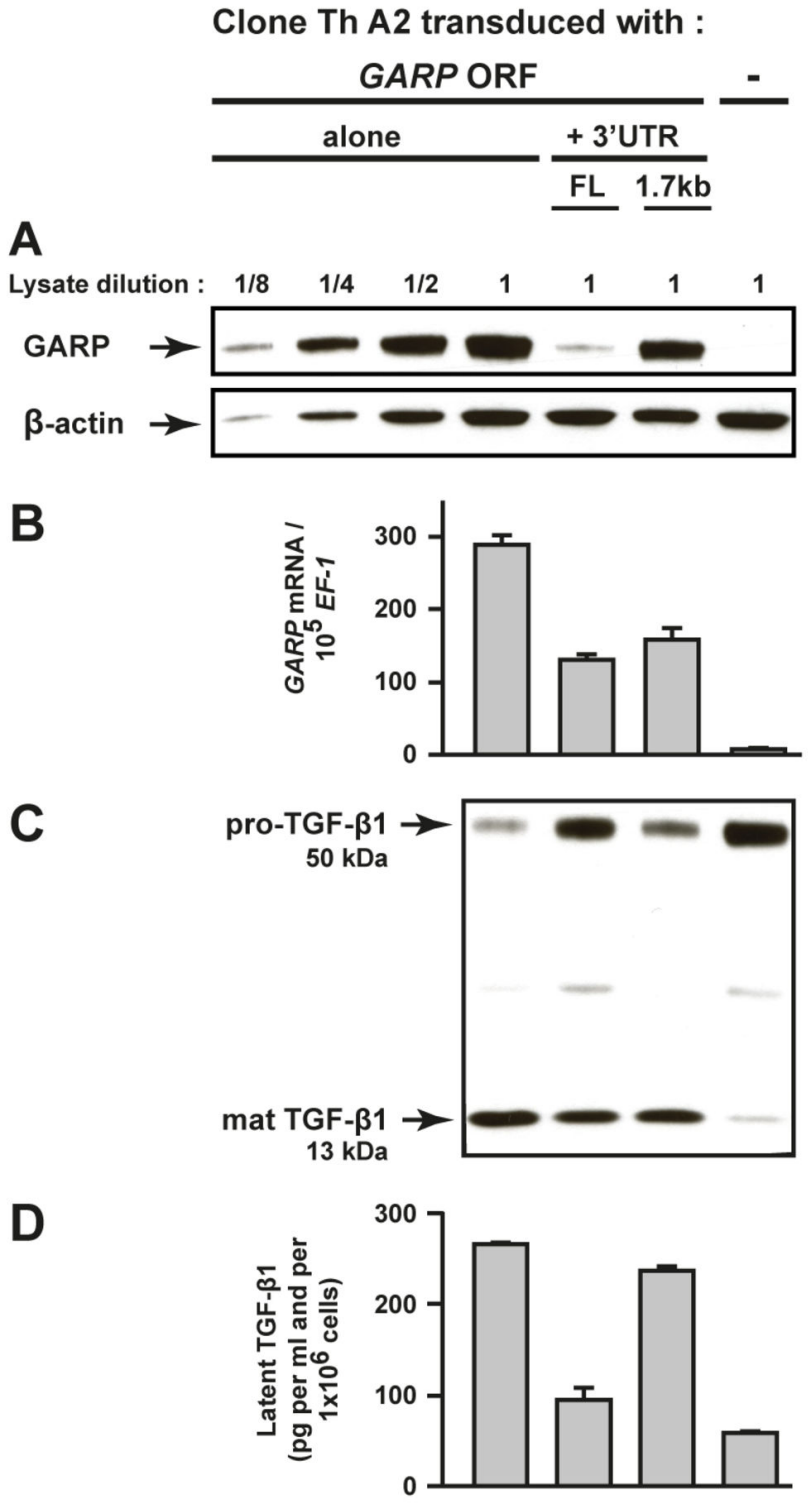
A short *GARP* 3’ UTR region controls GARP levels and production of TGF-β1 in T cells. Clone Th A2 was transduced with lentiviruses containing the GARP coding sequence (*GARP* ORF) alone, or followed by a full length (FL) or a truncated form (1.7 kb) of the *GARP* 3’ UTR. Transduced cells were restimulated for 24 hours with anti-CD3/CD28 antibodies in serum-free medium. **A to C**. Cell lysates were analysed by WB after SDS-PAGE in reducing conditions with antibodies against GARP, β-ACTIN (A) and a C-terminal epitope of TGF-β1 (C), and by RT-qPCR for *GARP* and housekeeping gene *EF-1* (B). Values in B are means of duplicates + SD. **D**. Concentration of latent TGF-β1 in the supernatants was measured by ELISA as indicated in Figure 2. Values are means of duplicates + SD, and are representative of 3 independent experiments. Similar results were obtained with unstimulated cells.

“antagomirs”). This could be due to low and variable transfection efficiencies, to incomplete inhibition of the miRNAs targeted by the antagomirs, or to as yet unidentified miRNAs targeting GARP in Th cells. We resorted to transducing a th clone with lentiviruses containing GARP ORF alone or followed by the full length or a 1.7 kb truncated form of the GARP 3’ UTR. The truncation removes the last 400 bp that contain the binding sites for miR-181a, b, c, d, miR-142-3p, and one of the two miR-185 sites (Figure 6B). Th cells transduced with the GARP ORF + 3’UTR full length expressed approximately 8 times less GARP protein than cells transduced with GARP ORF alone (Figure 9A). Truncation of the last 400 bp of the 3’ UTR restored GARP protein levels to those observed with GARP ORF alone (Figure 9A). We verified that GARP mRNA levels in cells transduced with the full length or truncated GARP 3’ UTR were similar, excluding that differences in GARP protein levels resulted from different levels of transgene expression (Figure 9B). Finally, decreased GARP levels in the presence of the full length 3’ UTR correlated with a reduced cleavage of pro-TGF-β1 (Figure 9C), and a reduced secretion of latent TGF-β1 (Figure 9D), by comparison to Th cells transduced with GARP ORF alone or fol

lowed by the truncated 3’ UTR.

Together, these results demonstrate that a 400-bp region of the *GARP* 3’ UTR, that is directly targeted by *miR-142-3p, miR-185* and the four *miR-181*, controls GARP protein levels and the amounts of TGF-β1 that are processed and secreted by human CD4^+^ T cells. 

## Discussion

Our results reveal a new regulation of TGF-β1 production in human T lymphocytes: GARP increases pro-TGF-β1 cleavage and latent TGF-β1 secretion, and these processes are controlled by miRNAs targeting the *GARP* 3’ UTR.

How could GARP contribute to pro-TGF-β1 cleavage, which depends on pro-protein convertase FURIN [27–29]? GARP expression does not appear to increase FURIN levels or activity (Figure 3A and 3B). But GARP may present pro-TGF-β1 to FURIN to facilitate its cleavage. In line with this possibility, we previously showed that GARP not only binds latent TGF-β1 but also pro-TGF-β1 [17], and the recently solved crystal structure of latent TGF-β1 showed that pro-TGF-β1 has the same conformation as latent TGF-β1 [24]. Alternatively, GARP may favor the production of properly folded latent TGF-β1. In this process, GARP could act in T cells as a chaperone, similarly to what was suggested for LTBPs in other cell types: formation of a disulfide linkage between LTBP, or GARP, and latent TGF-β1 could prevent anomalous disulfide bonds between LAP and the mature TGF-β1 peptide [30,31]. LTBPs are secreted proteins, and contribute to the deposition of latent TGF-β1 in the extracellular matrix. GARP, in contrast, is a transmembrane protein.

That GARP expression increased secretion of latent TGF-β1 by T cells was thus unexpected, even more so for the following reasons. First, GARP was shown to reduce the secretion of latent TGF-β1 by 293 cells, because it retained the cytokine on the cell surface through disulfide bonds [23]. Second, we observed that latent TGF-β1as disulfide-linked to GARP also in murine and human T cells. Therefore, we reasoned that latent TGF-β1 could be secreted as GARP/TGF-β1 complexes by T cells, but not by 293 cells, expressing GARP. Indeed, in the supernatants of T cells transfected with GARP, we found most latent TGF-β1 in high molecular weight complexes that also contained GARP. No such complexes were observed in the supernatant of GARP-transfected 293 cells. This suggests that GARP/TGF-β1 complexes are shed from murine and human T cell membranes by proteases that are absent or inactive on 293 cells. It also indicates that T cells may secrete different forms of latent TGF-β1 depending on the expression of GARP. Resting human Treg and Th clones secrete similar amounts of latent TGF-β1 [9] and do not express GARP. Upon TCR stimulation, Treg and Th clones increase latent TGF-β1 secretion similarly [9] but only Tregs express GARP [17]. Therefore, although the amounts of secreted latent TGF-β1 are similar, latent TGF-β1 complexed with GARP is produced only by stimulated Tregs. Some functions exerted by this new form of secreted latent TGF-β1 may differ from those of the previously described soluble latent TGF-β1, associated or not with LTBPs.

MicroRNAs are required for adequate development, differentiation and proliferation of effector CD4^+^ and CD8^+^ T lymphocytes, as revealed by the phenotype of mice with a T cell-specific deletion of *Dicer*, a gene coding an enzyme involved in the processing of most miRNAs [32,33]. Conditional deletion of *Dicer* in Tregs leads to lethal autoimmunity, indicating that miRNAs are also required for the development and functions of Tregs [34–36]. Tregs and Th cells have distinct miRNA expression profiles [37–40], but thus far, only a few individual miRNAs were shown to play a role in Treg or Th cell development or functions [41]. Murine *miR-155* and *miR-146*, for example, are expressed at higher levels in Tregs than in Th cells. Although *miR-155* was shown to contribute to the maintenance of Treg numbers through targeting of *SOCS1*, it is dispensable for proper Treg immunosuppressive function *in vivo* [42]. On the other hand, expression of *miR-146* in murine Tregs appears critical for the selective suppression of autoimmune Th1 responses, through targeting of *Stat1* [43]. Several miRNAs were also found down-regulated in murine or human Tregs versus Th cells [37,38,40,44]. Some were shown to target the 3’ UTR of *FOXP3* or *CTLA-4*, suggesting that low expression of given miRNAs is required for expression of genes important for Treg functions. Here we identified 6 miRNAs, namely *miR-142-3p*, *miR-185* and *miR-181a*, *b*, *c* and *d*, that are expressed at lower levels in human Treg than in Th clones, and that control GARP protein amounts through direct targeting of the *GARP* 3’ UTR. During the preparation of this manuscript, others also reported that *miR-142-3p* controls GARP levels in Tregs [45]. Fine-tuning of GARP levels by miRNAs will ultimately regulate the amounts of both soluble and membrane-bound latent TGF-β1 produced by Tregs. The soluble form can serve as a source for TGF-β1 activation by any cell type at distance, while the membrane-bound pool can be a reservoir for the production of active TGF-β1 close to the Treg surface, a feature we believe is important for the immunosuppressive function of these cells. Therefore, downregulation of *miR-142-3p*, *miR-185* and *miR181a*, *b*, *c*, *d* in the course of Treg cell differentiation might be required for the acquisition of a complete immunosuppressive phenotype.

Others have reported a reduced expression of *miR-142-3p* and *miR-181 b* and *d* in murine or human Tregs by comparison to Th cells [37,38,44,46]. While no functional consequence of the downregulation of *miR-181b* or *d* could be identified [38], the reduced expression of *miR-142-3p* was reported to allow increased Adenylyl Cyclase 9 activity and thus increase cAMP levels in murine Tregs [44]. Gap junction-dependent transfer of cAMP from Tregs to Th cells is thought to mediate part of the Treg suppressive functions [47,48]. Therefore, downregulation of *miR-142-3p* in Tregs may be important for the acquisition or maintenance of at least two immunosuppressive mechanisms.

## Materials and Methods

### Ethics statement

Experiments with human cells were approved by our Institution’s ethics committee (Commission d’Ethique Biomédicale Hospitalo-Facultaire de l’Université catholique de Louvain), under registration number B403201110966. Written informed consent for the use of blood samples was not always obtained, in accordance with the Belgian law of 19 December 2008 which states that, in the absence of written opposition by the patient, consent is considered given for residual body material. This applies to blood samples from hemochromatosis patients. No patient opposed the use of blood samples. Data obtained from blood samples were analyzed anonymously.

### Cells and transfections

Isolation and cultures of Treg and Th clones, and lentiviral transductions of human T cells were described previously (9,17).

Polyclonal cell populations enriched in Tregs (CD4^+^CD25^+^CD127^lo^ cells) and control Th cells (CD4^+^CD25^-^CD127^hi^ cells) were isolated from hemochromatosis donors in two steps: CD4^+^ T cells were enriched from blood with the RosetteSep Human CD4^+^ T Cell Enrichment Cocktail (StemCell Technologies), then separated into CD4^+^CD25^+^CD127^lo^ cells and CD4^+^CD25^-^CD127^hi^ cells on a flow cytometer (FACSAria, BD Biosciences). Sorted cells were used immediately (freshly isolated CD4^+^ T cells) or after *in vitro* amplification (expanded CD4^+^ T cells) during 12 to 14 days with anti-CD3/CD28 coated beads (Dynabeads Human T-Activator CD3/CD28, Life Technologies) in IMDM supplemented with 10% human serum, L-arginine, L-asparagine, L-glutamine, β-mercaptoethanol (5x10^-5^ M), methyl-tryptophane (200 µM) and IL-2 (300 IU/ml).

Freshly isolated or expanded CD4^+^ T cells were used in short-term stimulations or for miRNA mimic transfections. For the latter, 1.25x10^6^ cells resuspended in the “Unstimulated Human T Cells 4D-Nucleofector solution” with 2 to 2.5 µM of pre-miRNA precursors (Ambion) were electroporated with a 4D-Nucleofector instrument (Lonza).

We used “293T” cells, but refer to these cells as “293” cells throughout the manuscript to avoid confusion with T cells. 293 cells were transiently transfected with hGARP- and WT or C33S hTGF-β1-encoding plasmids using the TransIT-LT1 transfection Reagent (Mirus Bio).

A stable clone of murine BW5147.C2 cells expressing high levels of human GARP (hGARP) was derived by electroporation with a GARP plasmid and selection with puromycin under limiting dilution. The GARP-expressing clone was in turn electroporated with a plasmid encoding WT or C33S human TGF-β1 (kind gifts from Dr. J Keski-Oja, University of Helsinki, Finland). Clones expressing hGARP, and WT or C33S hTGF-β1 were selected with puromycin and geneticin under limiting dilution.

### Luciferase reporter assays

The *GARP* 3’ UTR was PCR amplified from a full-length cDNA clone (purchased from OriGene) and cloned downstream of the Renilla luciferase gene into the psiCHECK-2 vector (Promega). Mutagenesis of potential miRNA target sites was performed using the QuickChange II XL Site-Directed Mutagenesis Kit (Agilent Technologies). 293 cells were cotransfected in triplicate wells with 0,05 µg reporter plasmids and 10 nM miRNA mimics with Lipofectamine 2000 Transfection Reagent (both from Life Technologies) in a final volume of 75 µL. Renilla and Firefly luciferase activities were measured 24 hours after transfection with the Dual-Glo Luciferase Assay System (Promega).

### Quantification of cells with demethylated *FOXP3i1* sequences (Tregs)

Proportions of cells with demethylated *FOXP3i1* sequences were quantified in T cell populations by methyl-specific qPCR as described (9,11). Briefly, genomic DNA converted with sodium bisulfite was amplified by qPCR with primers and probes specific for the methylated or demethylated forms of FOXP3i1. As FOXP3 is located on the X chromosome, donor gender was taken into account to calculate the proportion of cells with a demethylated *FOXP3i1* allele.

### Immunoprecipitations (IP) and Western Blots (WB)

Anti-GARP and anti-LAP IPs were performed as described (17). Lysates, supernatants and IP products in Laemmli buffer supplemented or not with 5% β-mercaptoethanol (for reduced and non-reduced conditions, respectively) were submitted to SDS-PAGE and WB with the following primary antibodies: anti-GARP (Enzo Life Sciences, #ALX-804-867), anti-TGF-β1 (BD Pharmingen, #555052), biotinylated anti-LAP (R&D Systems, #BAF246), anti-FURIN (Enzo Life Sciences, #ALX-803-017) or anti-β-ACTIN (Sigma, #A5441). For the analysis of transfected BW5147 cell supernatants by WB (Figure 4B), similar results were obtained after ultracentrifugation (55000 rpm, 1 hour), indicating that the observed GARP/TGF-β1 complexes are not due to contamination by membranes.

### Short-term stimulations of T cells

Expanded T cells and T cell clones were stimulated with coated with anti-CD3 (Orthoclone OKT3, Janssen-Cilag, 1 µg/ml) and soluble anti-CD28 (BD Biosciences, 1 µg/ml). Freshly isolated T cells were stimulated with anti-CD3/CD28 coated beads (Dynabeads Human T-Activator CD3/CD28, Life Technologies). All short-term (24-48 hours) stimulations were performed in X-VIVO 10 serum-free medium (Lonza).

### TGF-β1 ELISA

Supernatants were left untreated or treated with acid, then analyzed by ELISA according to the manufacturer’s instructions (R&D Systems).

### RT-qPCR for miRNAs and *GARP* mRNA

For miRNA expression analysis, total RNA was extracted with the mirVana miRNA Isolation Kit (Ambion). Specific miRNAs were reverse transcribed and submitted to PCR on the ABI 7300 Real Time PCR System using the corresponding TaqMan MicroRNA Assay (Applied Biosystems). Results were normalized to the abundance of RNU44. RT-qPCR analysis of GARP mRNA was performed as previously described (17).

## Supporting Information

Figure S1 **Surface GARP and LAP expression on transfected 293 cells and T cells.** Cells transfected or transduced as indicated in Figure 2 were stained with anti-GARP or anti-LAP antibodies and analyzed by FACS. Clone Th A2 and Jurkat cells were analysed after stimulation with anti-CD3/CD28 antibodies.
(TIF)

Figure S2 **GARP expression and cleavage of the pro-TGF-β1 precursor in human Treg and Th cells.** Treg and Th cell populations used in Figure 4 were left resting or stimulated with anti-CD3/CD28 antibodies in serum-free medium. Cell lysates were collected after 24 hours and analyzed by SDS-PAGE under reducing conditions, followed by Western Blot with antibodies against GARP, β-actin and a C-terminal epitope of the TGF-β1 peptide.
(TIF)

Figure S3 **Six miRNAs that decrease GARP protein levels in transfected 293 cells.** 293 cells, which do not express detectable levels of endogenous GARP, were cotransfected with the indicated miRNA mimics and plasmids containing the GARP coding sequence alone (*GARP* ORF, right panels) or followed by the *GARP* 3’ UTR (*GARP* ORF + 3’UTR, left panels). Transfected cells were analyzed by WB with anti-GARP and anti-β-ACTIN antibodies. *miR-142-3p*, *miR-185* and *miR-181a* *to* *d* decreased GARP protein levels when cotransfected with the *GARP* plasmid containing the 3’ UTR, but had no effect in its absence. *miR-206*, which decreased luciferase reporter activity without reaching statistical significance (Figure 6A), did not decrease GARP protein levels.
(TIF)

Table S1 (EPS)
