# Supplementary figures and images for: GARP Is Regulated by miRNAs and Controls Latent TGF-β1 Production by Human Regulatory T Cells
Source: PLoS One. 2013 Sep 30;8(9):e76186. doi: 10.1371/journal.pone.0076186 (PMC3787020; doi:10.1371/journal.pone.0076186)

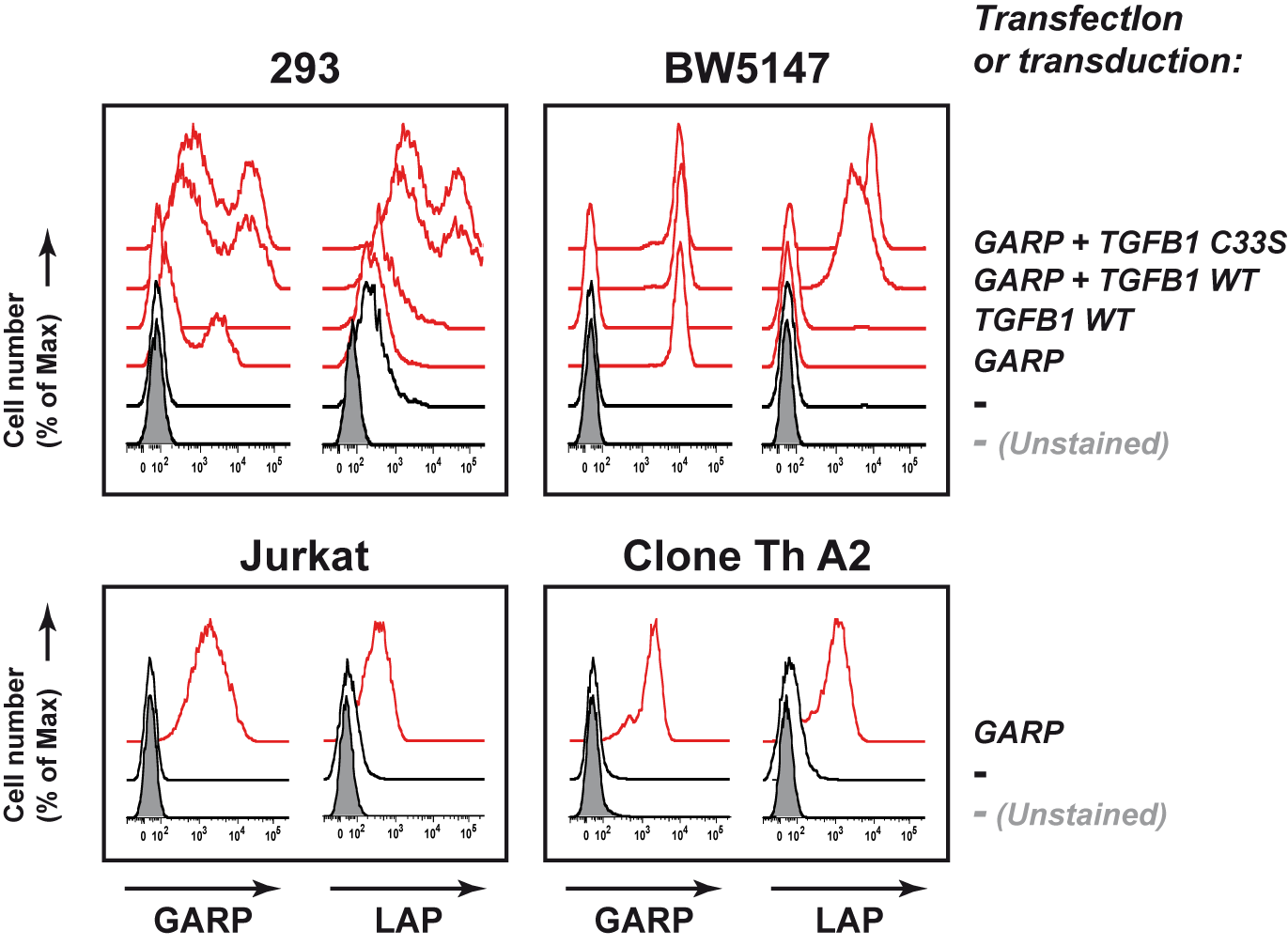

Supplement: Figure S1 — Surface GARP and LAP expression on transfected 293 cells and T cells. Cells transfected or transduced as indicated in Figure 2 were stained with anti-GARP or anti-LAP antibodies and analyzed by FACS. Clone Th A2 and Jurkat cells were analysed after stimulation with anti-CD3/CD28 antibodies. (TIF) [file pone.0076186.s001.tif]

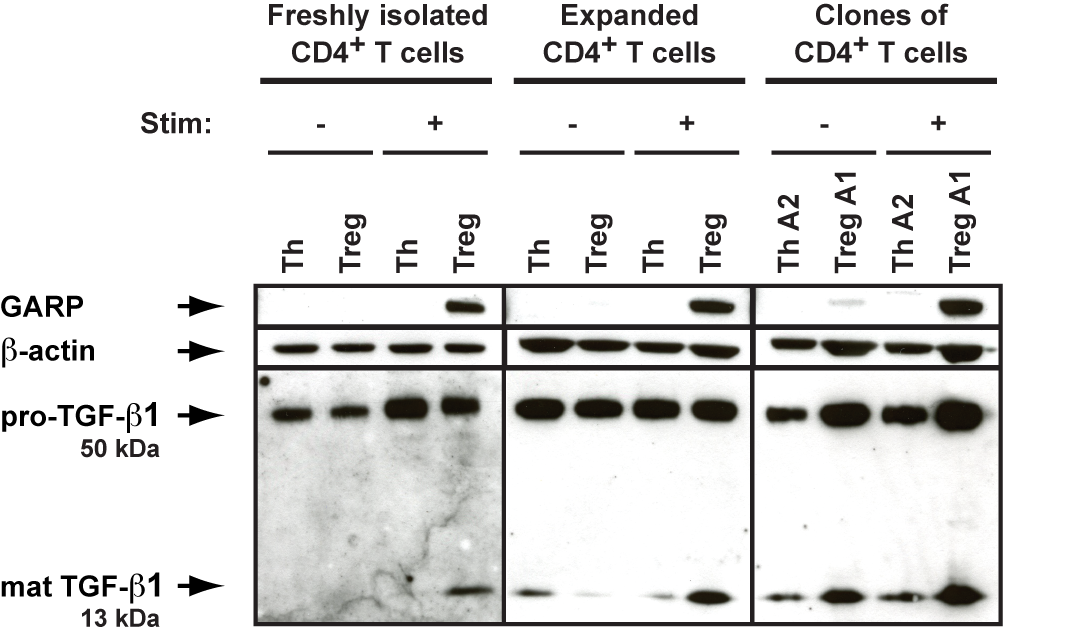

Supplement: Figure S2 — GARP expression and cleavage of the pro-TGF-β1 precursor in human Treg and Th cells. Treg and Th cell populations used in Figure 4 were left resting or stimulated with anti-CD3/CD28 antibodies in serum-free medium. Cell lysates were collected after 24 hours and analyzed by SDS-PAGE under reducing conditions, followed by Western Blot with antibodies against GARP, β-actin and a C-terminal epitope of the TGF-β1 peptide. (TIF) [file pone.0076186.s002.tif]

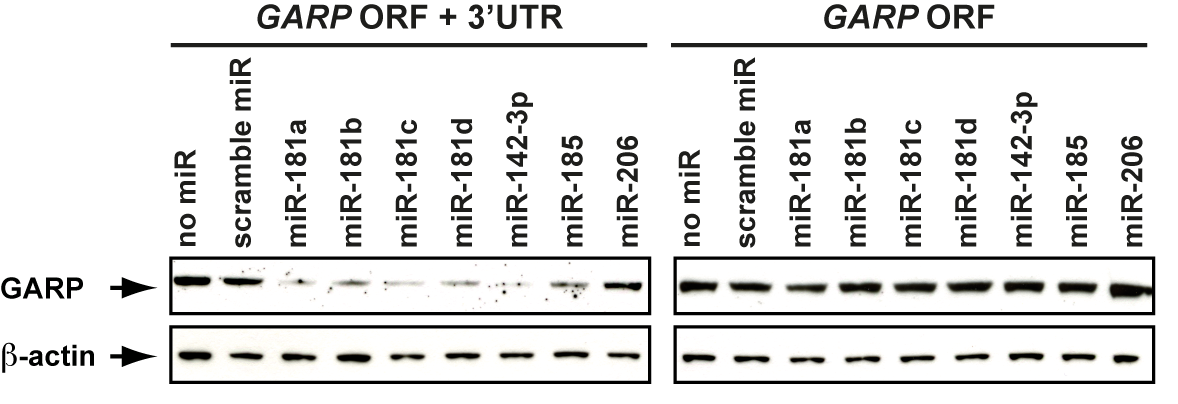

Supplement: Figure S3 — Six miRNAs that decrease GARP protein levels in transfected 293 cells. 293 cells, which do not express detectable levels of endogenous GARP, were cotransfected with the indicated miRNA mimics and plasmids containing the GARP coding sequence alone (GARP ORF, right panels) or followed by the GARP 3’ UTR (GARP ORF + 3’UTR, left panels). Transfected cells were analyzed by WB with anti-GARP and anti-β-ACTIN antibodies. miR-142-3p, miR-185 and miR-181a to d decreased GARP protein levels when cotransfected with the GARP plasmid containing the 3’ UTR, but had no effect in its absence. miR-206, which decreased luciferase reporter activity without reaching statistical significance (Figure 6A), did not decrease GARP protein levels. (TIF) [file pone.0076186.s003.tif]
